# Biomimetic manganese-eumelanin nanocomposites for combined hyperthermia-immunotherapy against prostate cancer

**DOI:** 10.1186/s12951-022-01248-5

**Published:** 2022-01-24

**Authors:** Yu Liu, Wenting Shang, Heng Liu, Hui Hui, Jun Wu, Wei Zhang, Pengli Gao, Kunxiong Guo, Yanli Guo, Jie Tian

**Affiliations:** 1 Beijing Advanced Innovation Center for Big Data-Based Precision Medicine, School of Medicine and Engineering, Beihang University, Beijing, 100191 China; 2 Department of Ultrasound, Southwest Hospital, Army Medical University, Chongqing, 400038 China; 3 CAS Key Laboratory of Molecular Imaging, Beijing Key Laboratory of Molecular Imaging, The State Key Laboratory of Management and Control for Complex Systems, Institute of Automation, Chinese Academy of Sciences, Beijing, 100190 China; 4 Department of Radiology, PLA Rocket Force Characteristic Medical Center, Beijing, 100088 China; 5 Key Laboratory of Big Data-Based Precision Medicine, Beihang University, Ministry of Industry and Information Technology, Beijing, 100191 China; 6 Engineering Research Center of Molecular and Neuro Imaging of Ministry of Education, School of Life Science and Technology, Xidian University, Xi’an, 710126 Shaanxi China

**Keywords:** Tumor-associated macrophages, Immunomodulation, Hyperthermia-immunotherapy, Eumelanin, Prostate cancer

## Abstract

Pro-tumoral and immunosuppressive M2-like tumor-associated macrophages (TAMs) contribute to tumor progression, recurrence and distal metastasis. However, current TAMs-modulating therapeutic strategies often encounter challenges including insufficient immune activation, weak antigen presentation ability and unsatisfactory antitumor immune performance. Herein, cyclic RGD peptide functionalized and manganese doped eumelanin-like nanocomposites (RMnMels) are reported for combined hyperthermia-immunotherapy against PC3 prostate cancer. The RMnMels could promote M2-to-M1 macrophage repolarization via scavenging multiple reactive oxygen species and remodeling the immunosuppressive tumor microenvironment. Following near-infrared light irradiation, RMnMels-mediated thermal ablation not only could destroy tumor cells directly, but also elicit the release of damage associated molecular patterns and tumor-associated antigens, provoking robust tumor immunogenicity and strong antitumor immune responses. The results showed that RMnMels could effectively scavenge reactive oxygen species and promote M2-to-M1 macrophage repolarization both in vitro and in vivo. Synergistically enhanced anti-tumor therapeutic efficacy was achieved following single administration of RMnMels plus single round of laser irradiation, evidenced by decreased primary tumor sizes and decreased number of distant liver metastatic nodules. The as-developed RMnMels may represent a simple and high-performance therapeutic nanoplatform for immunomodulation and enhanced antitumor immune responses.

**Graphical Abstract:**

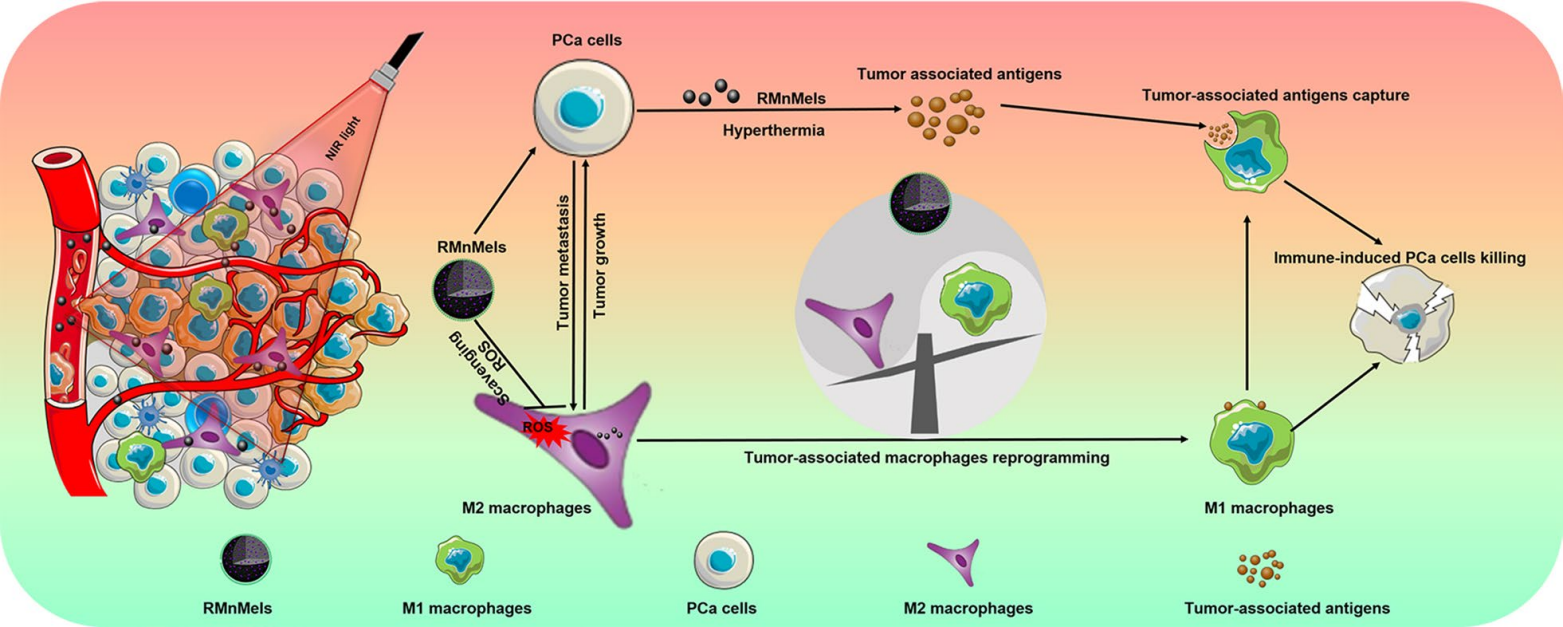

**Supplementary Information:**

The online version contains supplementary material available at 10.1186/s12951-022-01248-5.

## Introduction

Immunosuppressive tumor microenvironment (TME) is one crucial driver to promote tumorigenesis, progression, metastasis, immune evasion, and therapeutic resistance [1, 2]. As one of the most abundant components of immune cells infiltrating the TME, tumor-associated macrophages (TAMs) can be actively recruited to tumor sites and secrete various cytokines and chemokines, contributing to tumour immune evasion and immunotherapy failure [3–5]. TAMs are typically differentiated into anti-tumoral M1-like and pro-tumoral M2-like phenotype according to their polarization states [6, 7]. Usually, TAMs predominately present as M2-like phenotype, which is responsible for a variety of tumor-promoting activities at both primary and metastatic tumor sites [8–11]. Up to now, several TAMs-modulating therapeutic strategies have been explored as emerging paradigms [1, 2, 12]. However, one main drawback of depleting TAMs is that macrophages may lose their potential immunostimulatory effects as phagocytes and professional antigen-presenting cells [13]. It is of great interest to develop novel approaches for promoting the anti-tumor functional states of TAMs and enhancing the antitumor therapeutic efficacy.

It is well-known that TME is highly oxidative and characterized by overburdened reactive oxygen species (ROS) [14–16]. Previous studies revealed that ROS were crucial signaling molecules in M2 macrophage polarization and function [17]. M2 macrophages are more susceptible to ROS variation compared with M1 macrophages [18]. The M1 macrophages are effective effector cells for direct tumor cell killing through extensive release of various cytokines and local recruitment of pro-immunostimulating macrophages [13]. In this regard, selective M2-to-M1 macrophage repolarization has drawn significant attention for alleviating immunosuppression and improving antitumor responses [19, 20]. Recently, a variety of therapeutic agents (e.g., monoclonal antibodies, small molecular inhibitors, and complicated nanoparticles) have been extensively explored for M2-to-M1 macrophage repolarization [2, 17, 19–22]. Despite promising, these approaches encounter problems such as insufficient immune activation, weak antigen presentation ability, and unsatisfactory antitumor immune response [23, 24]. It is highly reasonable to integrate other appropriate synergistic strategies for improved therapeutic efficacy.

As an emerging therapeutic modality, photothermal monotherapy seems to be insufficient in complete tumor ablation attributed to the limited tissue penetration depth of light and the presence of immunosuppressive TME [25–27]. Recently, synergistic hyperthermia-immunotherapy has emerged as a novel regimen for expanding the attack area including residual tumor deep regions, disseminated metastasis and recurrent tumors [25, 28, 29]. In this scenario, local hyperthermia at tumor sites not only could ablate the primary tumor cells directly, but also could induce the release of multiple tumor-associated antigens and endogenous danger signals in situ, reversing the immunosuppressive TME, creating an immune-favorable TME, and strengthening the systemic antitumor immune responses [30–32]. However, the majority of the existing photothermal conversion agents are unable to be implemented in clinic attributed to their complicated and time-consuming fabrication procedures and possible long-term safety concerns [33]. Developing biomimetic nanoplatforms derived from naturally components via convenient procedures is highly beneficial [16, 34, 35], because they can be easily and gradually decomposed into nontoxic small molecular metabolic components [36]. Melanins are natural occurring biopolymers distributed in various parts of living organisms, possessing excellent biocompatibility and biodegradability [37, 38]. Owing to the abundance in reductive functional groups, melanins exhibit broad-spectrum radical scavenging capacities [37], which render them promising as novel potent antioxidative nanoplatforms.

**Scheme 1:**
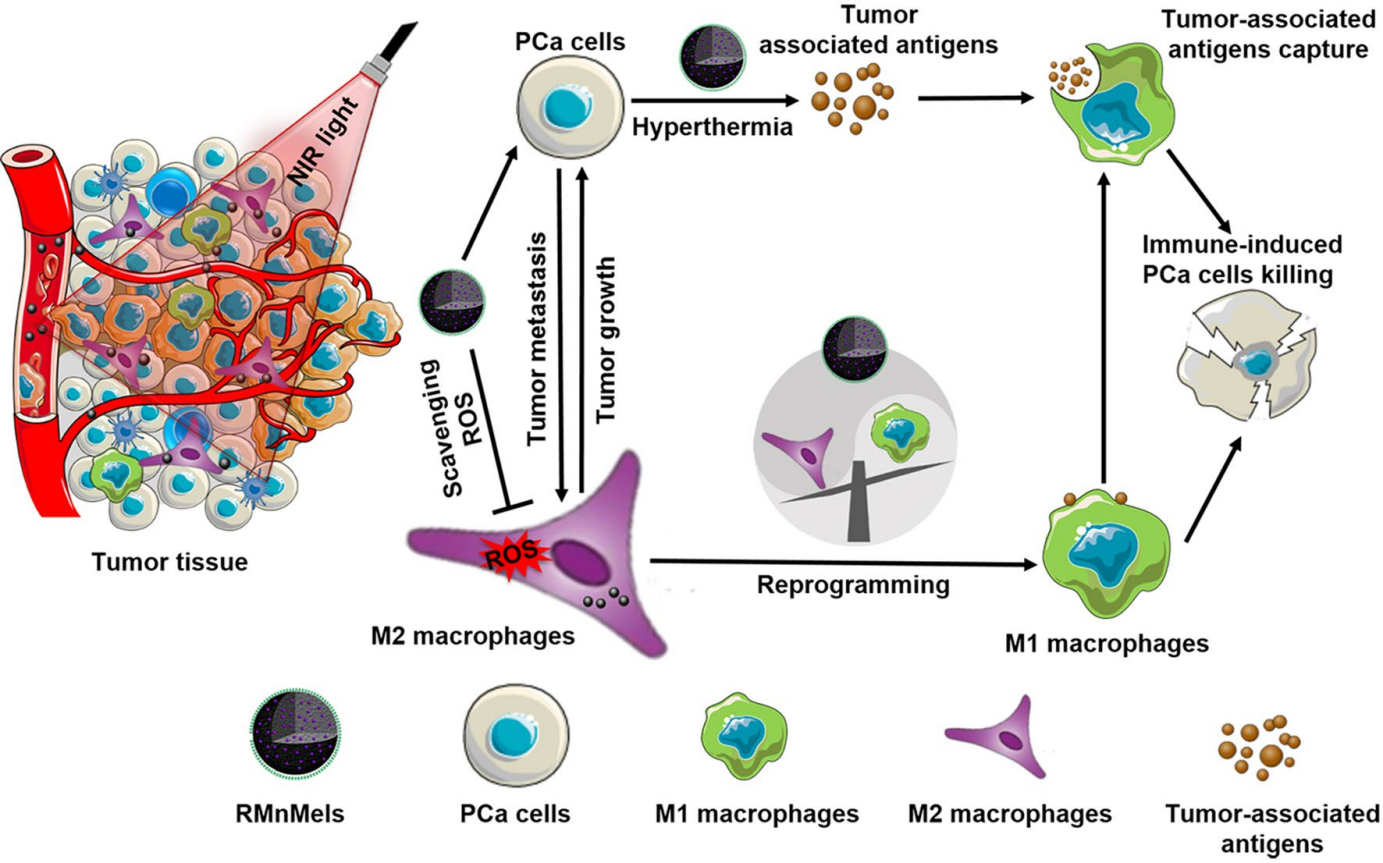
Schematic illustration of biomimetic RMnMels for enhanced prostate cancer therapy by reprogramming the immunosuppressive tumor microenvironment and synergizing hyperthermia therapy

Here, we report cyclic RGD peptide functionalized and manganese doped eumelanin nanocomposites (RMnMels) for synergistic hyperthermia-immunotherapy against PC3 prostate cancer (Scheme 1). The targeting peptide could facilitate preferential intratumoral accumulation of RMnMels via specific ligand-receptor interaction, realizing *T*_1_-weighted magnetic resonance/photoacoustic imaging for visualization of tumor in vivo. Then, RMnMels could promote M2-to-M1 macrophage repolarization via scavenging multiple ROS in situ in PC3 prostate cancer bearing mice. Notably, RMnMels-mediated local hyperthermia could destroy tumor cells directly, and the released damage associated molecular patterns and tumor-associated antigens could provoke robust tumor immunogenicity and strong antitumor immune responses. These together could synergistically reverse the immunosuppressive TME and realize improved therapeutic efficacy. As expected, single administration of RMnMels plus single round of laser irradiation realized remarkable anti-tumor efficacy, evidenced by decreased primary tumor sizes and decreased number of distant metastatic nodules. Taken together, the as-developed RMnMels represent a simple and high-performance therapeutic nanoplatform for immunomodulation and enhanced antitumor immune responses.

## Results and discussion

### Preparation and characterization of nanocomposites

**Fig. 1:**
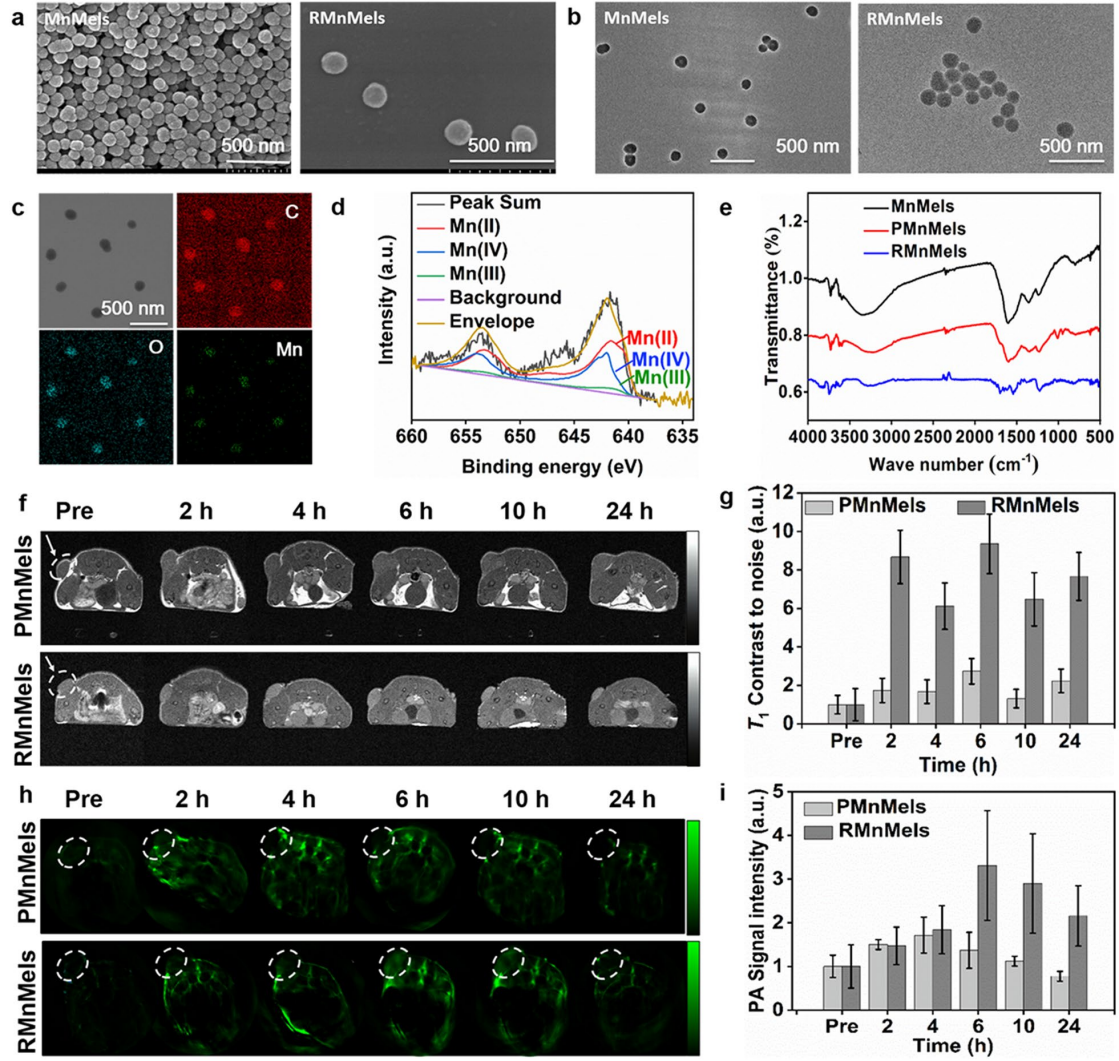
Characterization and imaging performance of nanocomposites. **a** SEM images. **b** TEM images. **c** Elemental mapping images. **d** High-resolution XPS spectra. **e** FT-IR spectra. **f** In vivo *T*_1_-weighted MR images of PC3 tumor-bearing mice prior to and at various time points post injection of nanocomposites. **g** Normalized *T*_1_ contrast-noise ratios within the tumor region. **h** In vivo photoacoustic images of PC3 tumor-bearing mice prior to and at various time points post injection of nanocomposites. **i** Normalized photoacoustic signal intensities within the tumor region

Manganese (Mn) doped eumelanin-like nanocomposites (MnMels) were first prepared through a simple intra-polymerization doping strategy. To improve the circulation stability and tumor targeting capacity of nanocomposites, PEGylated MnMels (PMnMels) and cRGD peptide modified MnMels (RMnMels) were synthesized [39]. Scanning electron microscopy (SEM) and transmission electron microscopy (TEM) images showed that both MnMels and RMnMels were uniform and monodispersed spherical in morphology, with a mean diameter of 80–100 nm (Fig. 1a, b). There was no apparent change in morphology between before and after RGD decoration. Energy dispersive X-ray (EDX) element mapping analysis showed homogeneous distribution of Mn element inside the entire nanostructures, confirming the successful doping of Mn element (Fig. 1c, Additional file 1: Fig. S1). The Mn loading efficiency was quantified as about 9.3% weight, determined by inductively coupled plasma mass spectrometry (ICP-MS). X-ray photoelectron spectroscopy (XPS) survey spectra showed that high-resolution Mn curve exhibited two binding energy peaks centered at 641.8 and 653.6 eV, which were assigned to Mn 2p3/2 and Mn 2p1/2 states, respectively. The peak centered at 646.0 eV was assigned to Mn 2p3/2 satellite peak (Fig. 1d, Additional file 1: Fig. S2). The nonlinear fitting analysis results showed that about 58% of manganese was in divalent state, 36.5% in quadrivalent state, and 5.5% in trivalent state, respectively. Dynamic light scattering (DLS) results showed that the mean hydrodynamic sizes of MnMels, PMnMels, and RMnMels were 122 ± 8.5 nm, 142 ± 17.5 nm, and 164 ± 11.5 nm, respectively, and all the zeta potentials of nanocomposites showed negative surface charge (Additional file 1: Fig. S3). Fourier transform infrared spectroscopy (FT–IR) results showed characteristic band signals for amide bond formation located at around 1650 cm^−1^ and 1535 cm^−1^, arising from the C=O and C–N stretching vibration (Fig. 1e). These results indicated successful synthesis of PMnMels and RMnMels.

Given the paramagnetic property of Mn ions, the feasibility of RMnMels as magnetic resonance (MR) contrast agents were evaluated. *T*_1_-weighted images demonstrated positive contrast enhancement in a concentration-dependent manner, and the longitudinal (*r*_1_) relaxivity was estimated to be 2.49 mM^−1^ s^−1^ (Additional file 1: Fig. S4). The MR contrast property of RMnMels was also confirmed by in vivo MRI (Additional file 1: Fig. S5). Time-resolved signal intensities in the kidneys and liver were monitored to evaluate the biodistribution and metabolic process of nanocomposites. After injection into PC3 prostate cancer bearing mice, both PMnMels and RMnMels provided positive contrast enhancement on *T*_1_-weighted images (Fig. 1f). Benefitted from peptide mediated active targeting, RMnMels accumulated at the tumor site more efficiently at 6 h post-injection, which was about 3.42 times higher than PMnMels (Fig. 1g). The concentration-dependent optical absorption of RMnMels (Additional file 1: Fig. S6) indicated their potential in photoacoustic imaging applications. As expected, RMnMels generated bright photoacoustic signals, which were in linearity with the mass concentrations (Additional file 1: Fig. S7). In accordance with MRI results, RMnMels showed more efficient intratumoral accumulation and produced stronger signal intensity at 6 h post-injection, which was about 2.4 times higher than that of PMnMels (Fig. 1h, i). ICP-MS results further confirmed the more effective efficient intratumoral accumulation of RMnMels (Additional file 1: Fig. S8). The results indicated great potential of RMnMels for *T*_1_-weighted MR/photoacoustic dual-modal imaging in vivo.

**Fig. 2:**
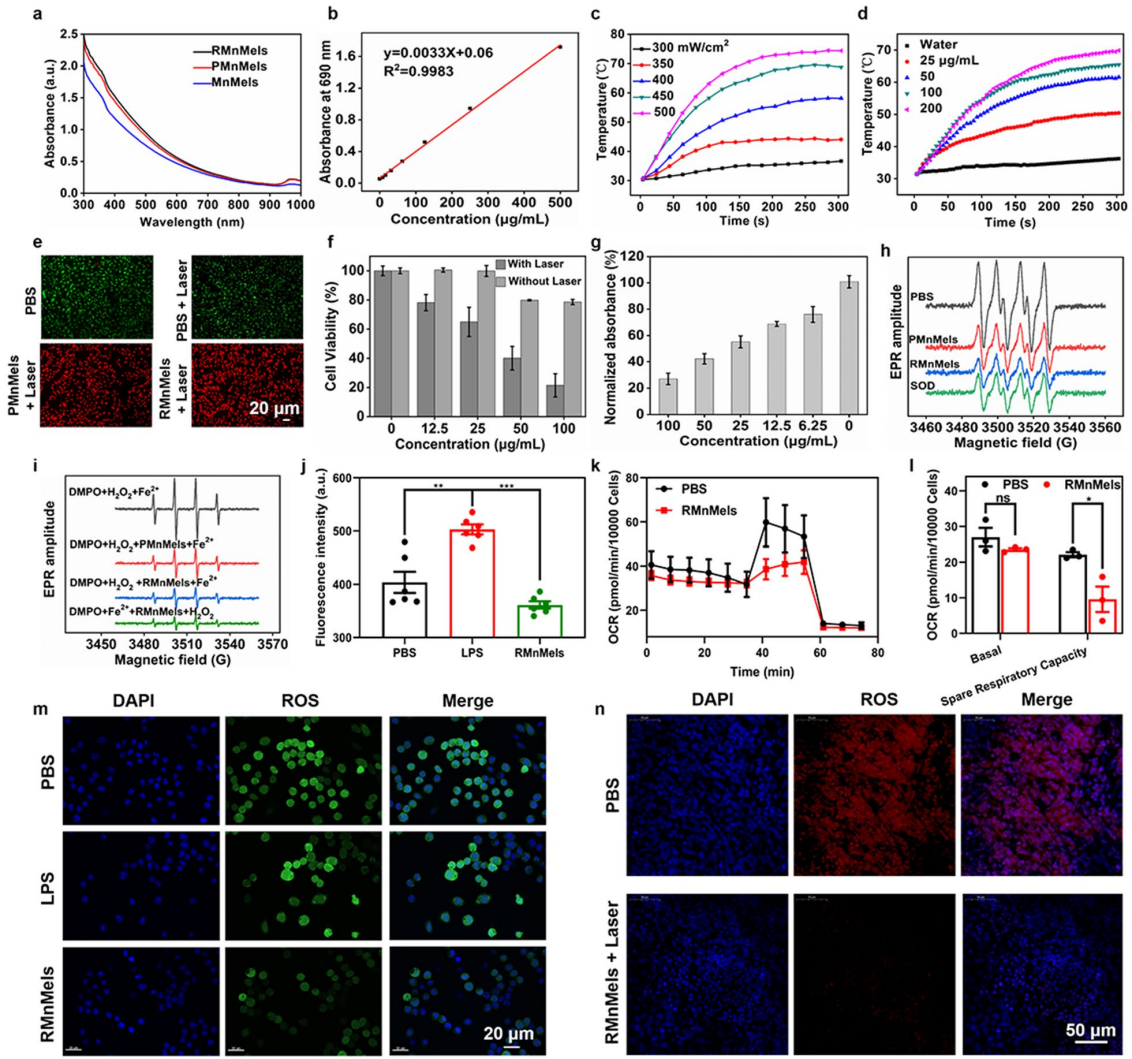
Optical absorption, photothermal properties, and free radical scavenging properties of nanocomposites. **a** UV–vis spectra. **b** The linear relationship between the optical absorbance at 690 nm of RMnMels and mass concentrations. **c** Photothermal heating curves of 200 µg/mL RMnMels during 690 nm laser irradiation with various laser densities. **d** Photothermal heating curves of RMnMels with various concentrations during 690 nm laser irradiation at 500 mW/cm^2^. **e** Calcein-AM/PI co-stained images of PC3 cells after different treatments. Scale bar, 20 μm. **f** PC3 cells viability after different treatments. DPPH (**g**), O^2**.**−^ (**h**), and ·OH (**i**) scavenging ability of nanocomposites. **j** ROS fluorescence intensity in RAW 264.7 cells after different treatments. **k** Effects of RMnMels on oxygen consumption rate of PC3 cells. **l** Quantification of basal respiration and spare respiratory capacity. **n** Fluorescence images for detection of intracellular ROS in PC3 tumor tissue slices. Scale bar, 50 μm. All experiments were run at least in triplicate. The statistical differences were quantified using Student’s t test (*p < 0.05, **p < 0.001, ***p < 0.0001)

Nanocomposites showed negligible cytotoxicity on PC3 and RAW 264.7 cells in the range of testing concentrations (Additional file 1: Fig. S9), indicating their biosafety and potential for in vivo applications. Optical microscope images clearly showed the binding of RMnMels with RAW 264.7 and PC3 cells (Additional file 1: Fig. S10). Cellular and tumor tissue TEM images showed that RMnMels were located in endosome-like structures (Additional file 1: Fig. S11), indicating efficient cellular internalization. Interestingly, cellular TEM images clearly showed that RMnMels could be internalized into lipopolysaccharide induced M1-like cells (Additional file 1: Fig. S12). Given that TME is characterized by elevated H_2_O_2_ levels, the H_2_O_2_-responsive degradation behavior of RMnMels was of great importance to minimize the poor biodegradation issue of many nanomaterials (Additional file 1: Fig. S13). The nanocomposites exhibited broad and monotonic optical absorption in the range of 300–1000 nm, and the absorbance at 690 nm was in good linear correlation with the mass concentrations (Fig. 2a, b). Benefiting from the strong near infrared (NIR) absorption, the temperature of RMnMels solution increased in laser power density/concentration dependent manners (Fig. 2c, d, Additional file 1: Fig. S14). Interestingly, RMnMels exhibited satisfactory photothermal stability (Additional file 1: Figs. S15, S16). The hyperthermia-mediated cell killing effect of RMnMels was verified by Calcein-AM/PI co-staining (Fig. 2e). Following RMnMels plus laser irradiation, remarkably decreased PC3 cell viability with increased incubation concentrations was determined. The cell viability results showed that the photothermal cell-killing efficacy was associated with nanoparticle concentrations (Fig. 2f).

RMnMels exhibited effective scavenging capacity against 2,2-di-(4-tert-octylphenyl)-1-picrylhydrazyl radical (DPPH, Fig. 2g), superoxide anion (O^2**.**−^, Fig. 2h) and hydroxyl radical (·OH, Fig. 2i). Following different treatments, RMnMels treatment resulted in the most significantly decreased intracellular fluorescence intensity in RAW 264.7 cells (Fig. 2j, m), indicating their effective ROS scavenging capacity. Tumor tissue immunofluorescence results showed that, RMnMels plus laser treatment induced more remarkably decreased ROS levels in the tumor tissues than PBS control (Fig. 2n). Given the excellent ROS scavenging capacity of RMnMels, their effect on mitochondrial function of PC3 cells was measured by a cellular energy metabolism analyzer. The results showed that RMnMels had a negative effect on oxygen consumption rate (OCR) compared with PBS control (Fig. 2k). PC3 cells co-incubated with RMnMels showed a decreased spare respiration by 12.53 ± 3.65% (P < 0.05, Fig. 2L). The results suggested that RMnMels could significantly inhibit mitochondrial function and alleviate the oxidative stress in PC3 cells.

### RMnMels promote M2-to-M1 macrophage repolarization

**Fig. 3:**
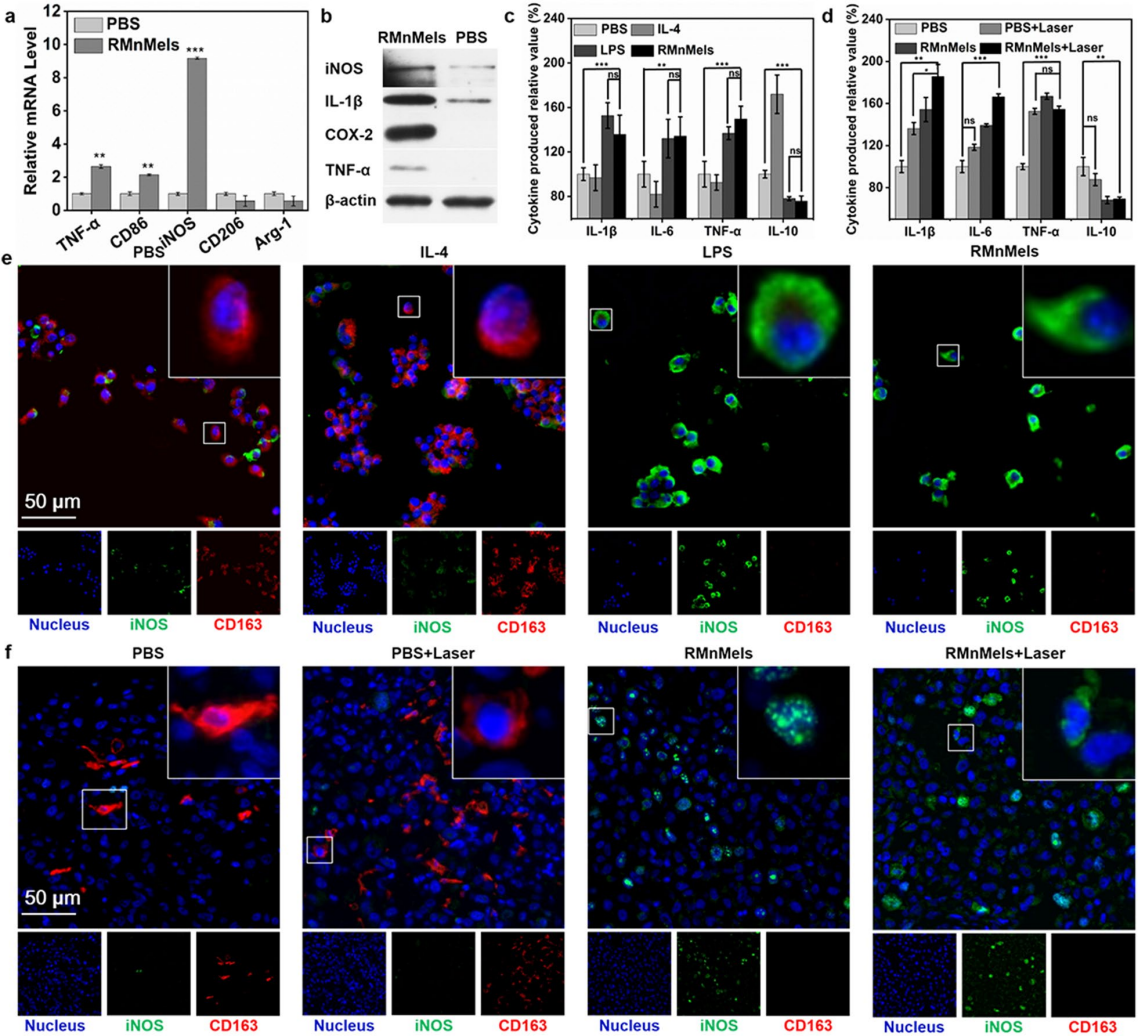
RMnMels for M2-to-M1 macrophages repolarization. **a** RT-PCR analysis of mRNA expression levels in RAW 264.7 cells. **b** Western blot analysis of protein expression levels in RAW 264.7 cells. ELISA assay for the levels of cytokines in RAW 264.7 cells (**c**) and PC3 tumor tissues (**d**) after different treatments. **e** Representative immunofluorescence images showing the phenotypes of macrophages after different treatments. Scale bars, 20 μm. **f** Representative immunofluorescence images of CD163 and iNOS in PC3 tumor tissue slices after different treatments. Scale bars, 20 μm. All experiments were run at least in triplicate. The statistical differences were quantified using Student’s t test (*p < 0.05, **p < 0.001, ***p < 0.0001)

Inhibiting M2 macrophages is considered as a promising cancer therapeutic approach [20]. The direct repolarization efficacy of RMnMels was analyzed using RAW264.7 cells. Real-time polymerase chain reaction (RT-PCR) results revealed that compared with PBS control, RMnMels treatment significantly up-regulated the mRNA levels of M1-related markers such as iNOS, TNF-α, and CD86, in which the iNOS increased as much as 10 times (Fig. 3a). Western blot assay showed that RMnMels treatment induced significant up-regulation of TNF-α, IL-1β and iNOS protein expression levels (Fig. 3b). Flow cytometric analysis showed that RMnMels treatment induced remarkably increased proportions of CD86^high^ M1 macrophages (Additional file 1: Fig. S17). Furthermore, the levels of inflammatory cytokines following different treatments in RAW 264.7cells were further verified by ELISA. The results showed that RMnMels treatment induced the upregulation of several M1 markers including IL-6, TNF-α, and IL-1β, and inhibited M2 marker IL-10 production (Fig. 3c). Cell immunofluorescence assay also showed that M1 marker iNOS expression increased and M2 marker CD163 expression decreased following RMnMels treatment (Fig. 3e). These results indicated that RMnMels were capable of driving the polarization of TAMs from M2-like toward M1-like phenotype.

### RMnMels alleviate immunosuppressive TME and significantly inhibit tumor growth

**Fig. 4:**
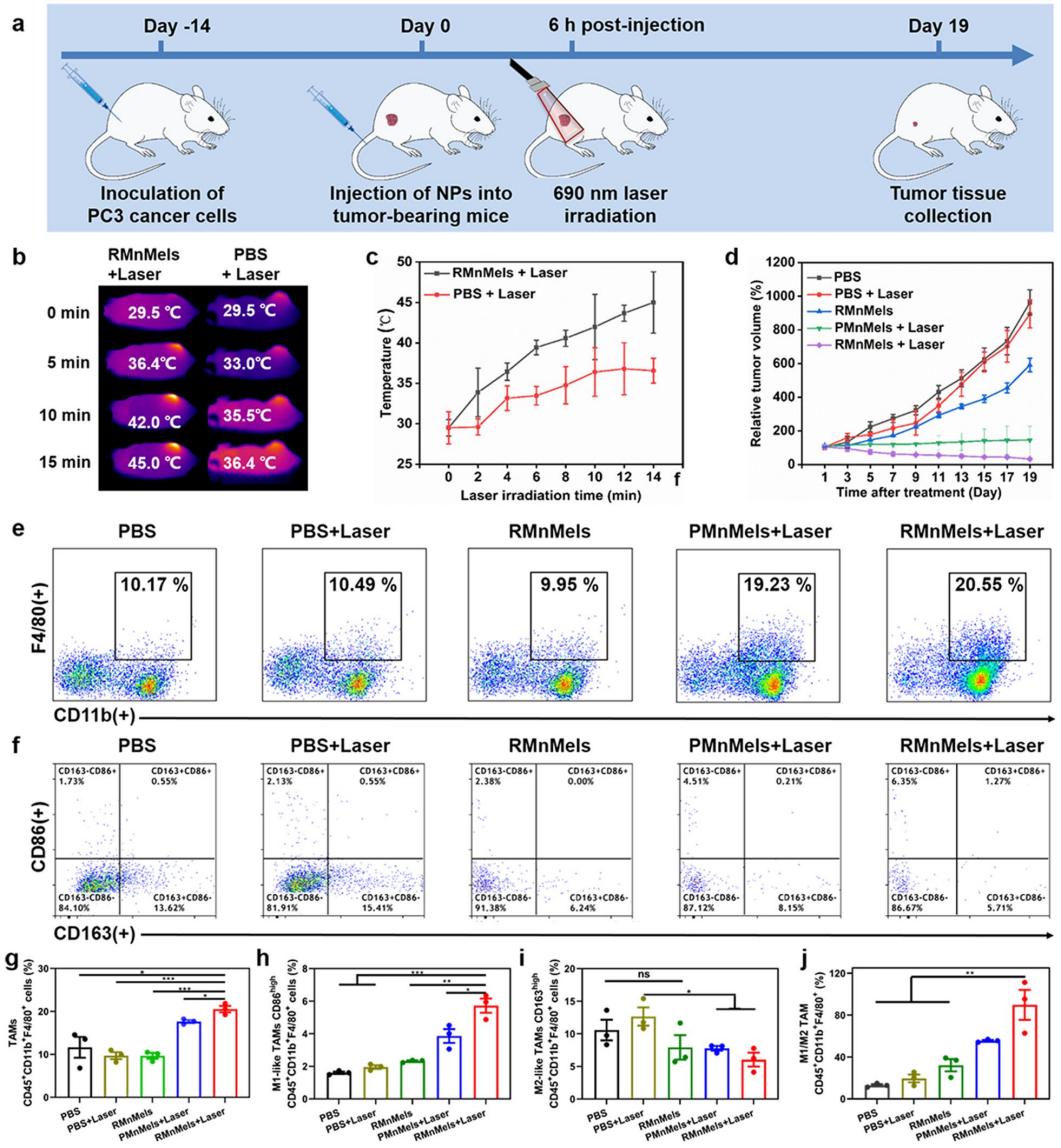
In vivo therapeutic evaluation of nanocomposites in PC3 tumor-bearing mice. **a** Schematic illustration of the time schedule for therapeutic treatment. Infrared thermal images (**b**) and temperature evolution curves (**c**) at tumor sites during different treatments. **d** Tumor growth curves during 19 days after different treatments. **e** The percentages of TAMs (CD45^+^CD11b^+^F4/80^+^) using flow cytometry. **f** The percentage of M1-like phenotype (CD86^high^ in CD45^+^CD11b^+^F4/80^+^ cells) and M2-like phenotype (CD163^high^ in CD45^+^CD11b^+^F4/80^+^) using flow cytometry. **g**–**j** Quantification of TAMs, M1-like TAMs, and M2-like TAMs and the ratio of M1/M2 TAMs infiltrated in tumor tissues by flow cytometry (n = 3). Data were presented as mean ± SD. The statistical differences were quantified using Student’s t test (ns, no significance, *p < 0.05, **p < 0.001, ***p < 0.0001)

The time schedule for treatment is shown in Fig. 4a. The immunological effects of thermal ablation was first assessed in PC3 tumor bearing mice. The temperature at the tumor sites increased to about 45.0 °C following RMnMels plus laser treatment. However, for mice receiving laser only, the temperature maintained at about 36.4 °C, which was insufficient to elicit any tissue damage (Fig. 4b, c). Besides directly destroying tumor cells, RMnMels-mediated hyperthermia could serve as an efficient immune stimulator to reverse the immunosuppressive TME and promote systemic antitumor immunological effects. Tissue immunofluorescence assay showed remarkably decreased M2 macrophages at the tumor sites following RMnMels and RMnMels plus laser treatment (Fig. 3f). Next, the serum levels of typical inflammatory cytokines were detected, given their roles as indicators of immunological responses. Consistent with cellular ELISA results, a similar shift in production of inflammatory cytokines in vivo was detected (Fig. 3d), which could contribute to enhanced immune responses. In the RMnMels only group, local hyperthermia effect was excluded due to the absence of laser irradiation, and the moderate changes of cytokine levels was attributed to RMnMels. Under the synergism of RMnMels and NIR irradiation, the IL-6, TNF-α and IL-1β levels further increased and remained within the normal ranges, indicating safe and efficient immunological responses. Collectively, these results revealed that RMnMels could significantly alleviate the immunosuppressive TME by inhibiting M2 macrophage polarization in vivo.

**Fig. 5:**
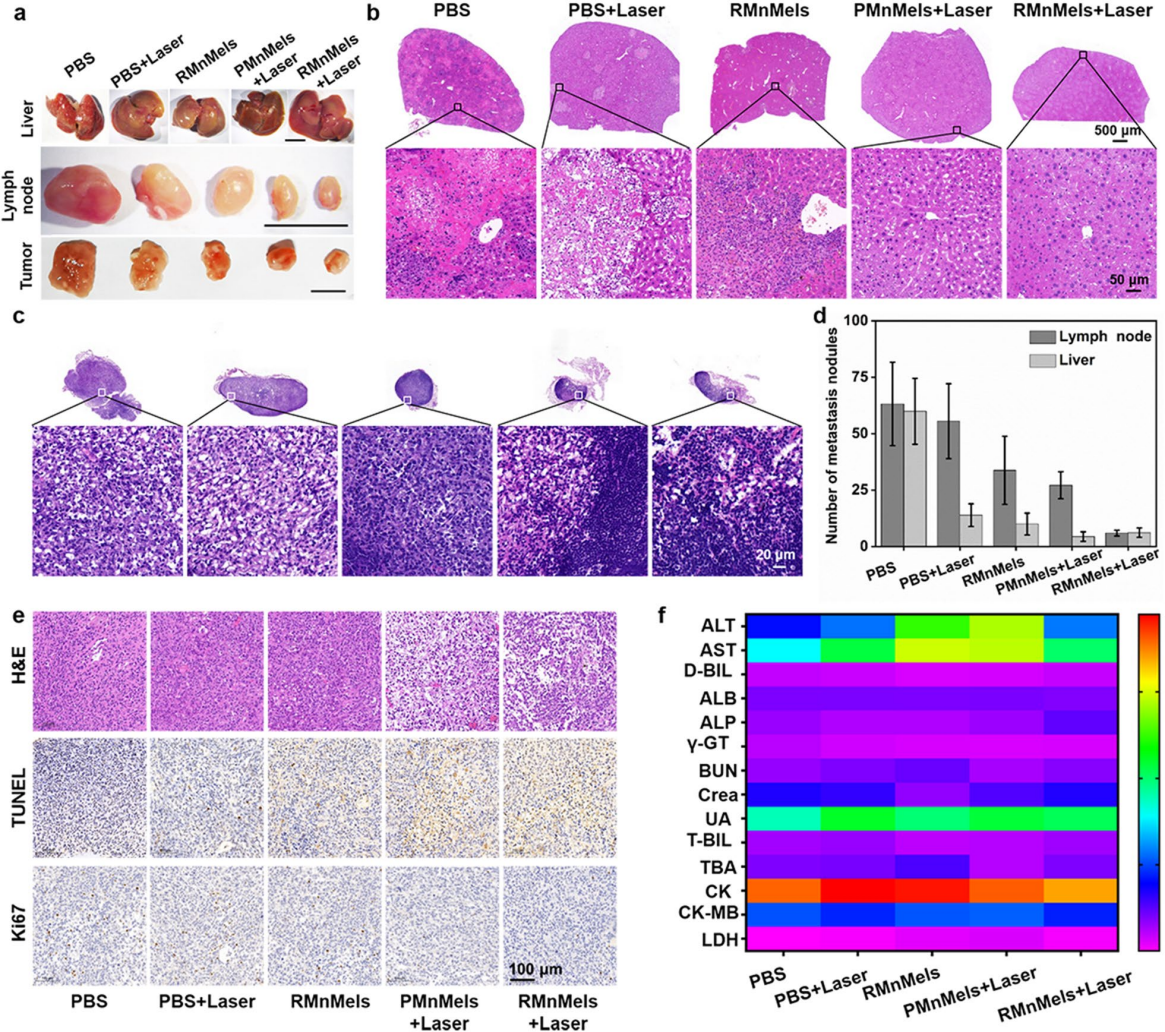
Histological analysis and safety evaluation. **a** Digital photographs of liver, tumor ipsilateral inguinal lymph nodes and tumor tissues in PC3 tumor-bearing mice on day 19 post treatment. Scale bars, 10 mm. Representative H&E-stained images of liver tissue slices (**b**) and inguinal lymph nodes (**c**) on day 19 post treatment. **d** Quantification of hepatic metastatic nodules and tumor ipsilateral inguinal lymphatic metastatic nodules on day 19 post treatment. **e** H&E, TUNEL and Ki67 stained images of tumor tissue slices on day 19 post treatment. Scale bar, 100 μm. **f** Serum biochemical indexes of tumor-bearing mice on day 19 post treatment

Then, the antitumor effect was evaluated during a period of 19 days (Fig. 4d, Additional file 1: Fig. S18). The antitumor effect was moderately ameliorated following RMnMels only treatment, attributed to their capacity in immunostimulation. PMnMels plus laser treatment had a similar antitumor effect because of inadequate intratumoral accumulation and subsequent restrained photothermal efficacy. Benefitted from specific tumor accumulation via peptide mediated active-targeting, RMnMels plus laser treatment showed maximized therapeutic efficacy. The synergism of immunostimulation and hyperthermia was evidenced by almost complete inhibition of tumor growth (Fig. 4d). To elucidate the antitumor mechanism of the prominent therapeutic performance, the profile of tumor-infiltrating immune cells was evaluated using flow cytometry (Additional file 1: Fig. S19). Compared with PBS only and PBS plus laser treatment, PMnMels plus laser treatment and RMnMels plus laser treatment significantly induced TAMs infiltration into tumor tissues (Fig. 4e, g). The RMnMels plus laser treatment significantly decreased the M2-like TAMs and increased the M1-like TAMs (Fig. 4f, h, i), resulting in an elevated M1/M2 ratio (Fig. 4j). The immunofluorescence and TUNEL staining also showed maximum M1-like TAMs and tumor cell apoptosis in the RMnMels plus laser group (Figs. 3f and 5e). Correspondingly, the immunosuppressive TME was remodeled in the RMnMels plus laser group.

### RMnMels significantly inhibit tumor metastasis and safety evaluation

In addition to significantly inhibited primary tumors, the minimum metastatic lesions in the liver and lymph nodules were observed in the RMnMels plus laser group (Fig. 5a–d). RMnMels plus laser treatment exhibited the most remarkable capability to inhibit tumor metastatic compared with other groups. Notably, H&E stained images (Additional file 1: Fig. S20) of major organs in PC3 tumor-bearing mice and serum biochemical both in tumor-bearing mice and healthy mice (Fig. 5f, Additional file 1: Fig. S21) showed scarcely any abnormality. The results indicated satisfactory safety of RMnMels, which was suitable for in vivo bio-applications.

### Limitations

This study has limitations. First, RMnMels are reported as a simple and high-performance therapeutic nanoplatform for combined hyperthermia-immunotherapy against PC3 prostate cancer, in which RGD peptide served as a well-acknowledged targeting module for improved delivery of nanocomposites. However, whether RGD-integrin specific recognition itself has potential biological effect lacks methodologically acceptable evidence, which may be verified by using a scrambled peptide or a blocking experiment. Second, as a relatively simplified model for the proposed concept, human prostate cancer cell line PC3 based subcutaneous heterotopic tumor model was established in nude mice in this study. However, it could not objectively simulate the real physio-pathologic characteristics of the primary human tumors. Orthotopic patient-derived xenografts models in humanized mice, or animal-originated cell line-based orthotopic tumor models in mice with a normal immune system should be established for assessing the theranostic performance of RMnMels, which is one potential preclinical direction in the future.

## Conclusions

In summary, an immunomodulation-enhanced nanoplatform is constructed to realize synergistic hyperthermia-immunotherapy. Unlike conventional TAMs-modulating therapeutic strategies, the intrinsic nature of melanin endows the as-developed RMnMels with satisfactory biocompatibility and multifunctionality, and they could scavenge excess ROS and induce M2-to-M1 macrophage repolarization at tumor sites in situ, remodeling the immunosuppressive TME effectively. Following thermal ablation induced by local light, RMnMels could serve as immunological stimulators to improve the immunostimulatory efficacy and strengthen the antigen presentation ability. Our results demonstrated that high-performance hyperthermia-immunotherapy synergism was achieved following single administration of RMnMels plus single round of laser irradiation. This work will open up new insights for enhanced antitumor immune responses and improved tumor therapeutic efficacy.

## Experimental section

### Materials

Potassium permanganate (KMnO_4_), 3,4-dihydroxy-dl-phenylalanine (dl-DOPA), propidium iodide (PI), and calcein acetoxymethyl ester (Calcein AM) were purchased from Aladdin Reagent (Shanghai, China). Thiol-terminated methoxy-poly (ethylene glycol) (mPEG-SH, molecular weight = 5000 Da) was purchased from Seebio (Shanghai, China). Cyclic Arg-Gly-Asp (cRGD) peptide was purchased from ChinaPeptides Co., Ltd. (Jiangsu, China). Minimum essential medium (MEM), Dulbecco’s modified Eagle’s medium (DMEM), and fetal bovine serum (FBS) were purchased from Gibco (New York, USA). 2,2-Di-(4-*tert*-octylphenyl)-1-picrylhydrazyl radical (DPPH) was purchased from Tokyo Chemical Industry (Tokyo, Japan). IL-4 were purchased from BioLegend (San Diego, USA). Lipopolysaccharide (LPS), ammonium hydroxide (30%), β-actin, and HRP-conjugated mouse monoclonal antibody were purchased from Sigma-Aldrich (St. Louis, USA). ELISA assay kits (TNF-α, IL-6, IL-10, IL-1β) were purchased from MeiBiao Biological Technology (Jiangsu, China). CCK-8 assay kit was purchased from Beijing Solarbio Science & Technology (Beijing, China). ROS assay kit was purchased from Beyotime (Shanghai, China). Anti-CD163 and anti-iNOS were purchased from Abcam (Massachusetts, USA), CD86-PE and CD206-APC monoclonal antibodies were purchased from eBiosciences (San Diego, USA). COX-2 murine polyclonal antibody and Arginase-1 (Arg-1) murine monoclonal antibody was purchased from Proteintech (Chicago, USA).

### Synthesis of MnMels, PMnMels and RMnMels

Manganese doped eumelanin-like nanocomposites (MnMels) were prepared through a facile and simple intra-polymerization doping strategy as we previously described [40]. For preparing PEGylated MnMels (PMnMels), MnMels and mPEG-SH (feeding mass ratio = 1:5) were vigorously stirred in pH = 10 buffer solution for 12 h at room temperature. The as-obtained PMnMels were purified by several centrifugation-redispersion processes (17,500 rpm, 15 min) to remove residual mPEG-SH. The cRGD peptide modified MnMels (RMnMels) were prepared via a Michael addition approach. Briefly, 2 mg/mL MnMels was added in 950 µL NH_3_·H_2_O (30%) solution (pH = 10). Then, 500 nmol cRGD in 50 µL water was added and reacted at 4 °C for 12 h. The products were retrieved by four times of centrifugation-redispersion processes (17,500 rpm, 15 min) to remove excess peptide. Finally, the as-obtained RMnMels were re-dispersed in deionized water for subsequent experiments.

### Physicochemical characterization

The shape and morphology of nanocomposites were acquired by transmission electron microscopy (TEM, Tecnai G2 F30 S-TWIN, USA) and scanning electron microscope (SEM, Hitachi SU-70, Tokyo, Japan). Elemental mapping scanning images was acquired using energy-dispersive X-ray (EDX) spectroscopy equipped on TEM. The zeta potential and hydrodynamic sizes of nanocomposites were determined by dynamic light scatterer (DLS, Zetasizer Nano ZS90, Malvern Instruments, UK). The chemical functional groups were measured by Fourier transform infrared spectroscopy (FTIR, Nicolet iS10, USA). The valence states of manganese component were analyzed by X-ray photoelectron spectrometer (XPS, Thermo Fisher Scientific, USA). The metal ion contents in nanocomposites were determined by inductively coupled plasma mass spectrometry (ICP-MS, PerkinElmer, USA, Agilent 7800). The optical absorption profile in the UV–vis region was acquired using an UV–vis spectrophotometer (Shimadzu Company, Japan). All electron spin resonance (ESR) spectra measurements were acquired by an ESR spectrometer (Bruker A300-10/12, Germany) at ambient temperature.

### Magnetic resonance imaging

To verify the MR contrast capacity of RMnMels, relaxivity measurement was carried on a MR apparatus (Bio-Spec, Bruker, Karlsruhe, Germany). RMnMels with various metal molar concentrations (0-1 mM) were dispersed in ultrapure water. Phantom images were acquired at room temperature. The *r*_1_ relaxivity was determined by a linear fitting function between 1/relaxation time (s^−1^) and manganese ion molar concentrations (mM). In vivo* T*_1_-weighted MR images were acquired on a MR apparatus (M3TM, Aspect Imaging, Israel). The parameters were set as follows: TR/TE: 500/12 ms; slice thickness: 0.8 mm; field of view: 3.0 × 6.0 cm; matrix: 256 × 128. During image acquisition, all mice were anesthetized using a mixture of isoflurane and compressed air.

### Photoacoustic imaging

3 mL RMnMels with various concentrations (0–500 µg/mL) were placed in a cylindrical model for photoacoustic signal detection, using a multispectral photoacoustic tomography imaging system (MSOT, iTheraMedical, Germany). The parameters were set up to allow absorbance of 690–900 nm. The photoacoustic signal intensity was quantitatively analyzed.

### Biodistribution of RMnMels in vivo

200 µL RMnMels solution (12 mg/kg body weight) was intravenously injected into PC3 tumor bearing mice. *T*_1_-weighted MR images were acquired prior to and at various time points post injection of RMnMels using a MR scanner. To quantify the contrast enhancement, the signal intensity was determined by finely analyzing ROI of the images. To eliminate possible variations among mice, normalized *T*_1_ relaxation times were analyzed against post-injection time and by setting that of prior to injection as 1.0. The photoacoustic signals in the tumor regions were acquired prior to and at various time points post injection of RMnMels using MSOT system, with images acquired in 8 min with 0.5 mm per step.

### Photothermal conversion property

To measure the photothermal conversion property of RMnMels, 200 µL RMnMels solution with various concentrations (0–200 µg/mL) underwent exposure to a 690 nm laser (500 mW/cm^2^, 300 s). And 200 µg/mL RMnMels aqueous solution were exposed to a 690 nm laser at various laser power densities for 300 s. The infrared thermal images were acquired and the temperature variations were monitored using an infrared thermal camera (FLIR E75-42 Advanced Thermal Imaging Camera, USA).

### Multi-antioxidative activities of RMnMels

For DPPH scavenging assay, 200 µg/mL DPPH solution in ethanol was mixed with various concentrations of RMnMels (0–100 µg/mL) solution with 1:1 volume ratio. After kept 30 min away from light at room temperature, the optical absorbance at 516 nm was determined. The spin-trap free radical scavenging DEPMPO was used as the trapper for O_2_^**.**−^ and ·OH radicals. The stock solution of O_2_^**.**−^ radicals was produced at room temperature. DMSO containing saturated air was fully mixed with 5 mmol/L NaOH solution and 1% H_2_O. After fully stirred and placed for 30 min, PBS solution containing EDTA (0.2 mM) and DMPO (40 mM) was added. ESR test was carried out at 77 K. For electron paramagnetic resonance (EPR) measurements, DMSO solution was added into a water solution containing EDTA (0.2 mM), DEPMPO (40 mM), and PBS/PMnMels/RMnMels/SOD, and the EPR spectra were recorded. ·OH was measured using an EPR spectra spin-trapping method with DMPO-OH as the spin-trapping agent in the condition of absence or presence of nanocomposites. The ·OH was produced by a Fenton reaction between Fe^2+^ ions and H_2_O_2_. Then, nanocomposites were added into the mixture of DMPO and H_2_O_2_, followed by Fe^2+^ ions addition. Last, RMnMels were pre-incubated with DMPO and Fe^2+^, followed by H_2_O_2_ addition.

### The effect of RMnMels on mitochondrial function

The effect of RMnMels on mitochondrial function was measured by Seahorse XFp Extracellular Flux Analyzer (Agilent Technologies, USA). All experiments were performed according to the manufacturer’s protocol. Seahorse XFp Cell Mito Stress Test Kit (Agilent Technologies) were used to measure oxygen consumption rate (OCR), basal respiration (Basal Res), and spare respiratory capacity (Spare Res Cap). Briefly, 2 × 10^4^ PC3 cells were plated per well and seeded in a Seahorse XFp cell culture microplates and incubated for 12 h at 37 °C, respectively. 50 µg/mL RMnMels were added and co-incubated with cells for another 24 h. Untreated cells served as negative control. The results were normalized to cell number. Data were analyzed using Seahorse XFp Wave software.

### Reactive oxygen species (ROS) levels in vitro

RAW 264.7 cells were first pre-stimulated with PBS, 100 ng/mL LPS and 50 µg/mL RMnMels for 24 h, respectively. Then, cellular ROS levels were detected using a DCFDA-Cellular ROS Detection Assay Kit (Solarbio, Beijing, China). According to the instructions of the manufacturer, 10 µM 2′,7′-dichlorofluorescin diacetate (DCFDA) was added at 37 °C for 30 min, and fluorescence was measured using a confocal laser scanning microscopy (Andor Dragonfly, UK).

### Cell experiments

Human prostate cancer cell line PC3 and RAW 264.7 cells were obtained from the Cell Culture Collection of Chinese Academy of Sciences (Shanghai, China). PC3 and RAW 264.7 cells were maintained in MEM and DMEM containing 10% FBS and 1% penicillin/streptomycin, respectively. The cells were kept in a homothermal cell incubator containing 5% CO_2_ at 37 °C.

### For cytotoxicity assay

PC3 and RAW 264.7 cells were seeded in 96-well cell culture plates (1 × 10^4^ cells/well) and incubated overnight at 37 °C, respectively. RMnMels with various concentrations were added and co-incubated with cells for another 24 h. A standard CCK8 assay was carried out to assay the cell viability. For in vitro photothermal cytotoxicity, PC3 cells were seeded in a 12-well cell culture plate (5 × 10^4^ cells/well) and incubated overnight at 37 °C. The cells were co-incubated with 100 µg/mL PMnMels and RMnMels for 12 h, respectively. Then, cells were washed for several times and replaced with fresh medium, following by exposure to a 690 nm laser at 500 mW/cm^2^ for 5 min. After incubation for another 4 h, Calcein AM and PI co-staining was performed for 30 min, and then fluorescence images were acquired. To quantify the photothermal cytotoxicity of RMnMels, PC3 cells were seeded in a 96-well cell culture plate (1 × 10^4^ cells/well), and co-incubated with various concentrations of RMnMels for 12 h. Then, cells were exposed to a 690 nm laser at 500 mW/cm^2^ for 5 min, washed for several times and then incubated in fresh cell culture media for another 4 h. A standard CCK8 assay was carried out to assay the cell viability.

### Cellular uptake and degradability assay

For cellular uptake experiment, PC3 cells were co-incubated with 50 µg/mL RMnMels for 12 h. Then, cells were washed for several times and optical microscope images were acquired. RAW 264.7 cells were co-incubated with various concentrations of RMnMels for 12 h, then visualized by an optical microscope. For bio-TEM, cells were harvested by trypsin digestion and centrifugation, fixed in 2.5% cold glutaraldehyde, dehydrated, embedded, sectioned, and stained for TEM observation. To investigate the internalization of RMnMels by M1-like macrophages, RAW 264.7 cells were first co-incubation with 100 ng/mL lipopolysaccharide for 24 h, and the polarization state of cells were verified by flow cytometry. After co-incubation with 50 µg/mL RMnMels for 12 h, the cells were washed for several times for TEM observation. To evaluate the degradation profiles in vitro, 100 µg/mL RMnMels were suspended into PBS (pH = 7.4) without or with H_2_O_2_ (2.5 mM), respectively. The solution was kept stand at room temperature. At selected time intervals, digital photos of the solutions were taken, and then 100 µL solution was collected for detecting the accumulated degradation content using an UV–vis spectrometer. TEM images were obtained for intuitive observation of time-dependent structural evolution during degradation process.

### The effect of RMnMels on M2 macrophage polarization in vitro

RAW264.7 cells were first pre-stimulated with 100 ng/mL IL-4 for 24 h to obtain M2-like phenotype, and then co-incubated with or without 10 µg/mL RMnMels for another 24 h. The supernatants were carefully harvested for measuring TNF-α, IL-6, IL-1β, and IL-10 levels using ELISA assay kits (R&D Systems, USA). Meanwhile, the cells were collected for determining the gene expression levels of CD86, TNF-α, iNOS, Arginase-1 and CD206 by reverse transcription-polymerase chain reaction (RT-PCR). The primer sequences used are shown in Additional file 1: Table S1. The gene expression level were normalized to the housekeeping gene reduced glyceraldehyde-phosphate dehydrogenase (GAPDH). For determining the protein expression levels of iNOS, IL-1β, COX-2 and TNF-α, western blot assay was carried out, with β-actin as the internal reference. To detect macrophage surface markers, cells were labelled with CD86-PE and CD206-APC mouse monoclonal antibodies. For each sample, at least 3 × 10^4^ cells were run on a flow cytometer and data was analyzed using FlowJo software (BD Bio-sciences, USA).

### Flow cytometry assay of cells in tumor tissues

Tumor tissues were collected and first digested into single-cell suspension at 37 °C, and then filtered with a 70 μm cell screen. Cells were collected centrifugally and dispersed in PBS containing 1% FBS. Then, cells were stained with 1 µL Fixable Viability Stain 510 (*FVS510*) (BD Horizon™) for 15 min in 1 mL PBS at 4 °C to distinguish live and dead cells. Cells were then washed and stained with 0.5 µg anti-mouse CD16/32 for 10 min at 4 °C. Then, the cells and were stained with APC-Cy7-labelled anti-mouse CD45, APC-Cy5.5-labelled anti-mouse CD11b, Alexa Fluor 488-labelled anti-mouse F4/80, APC-labelled anti-mouse CD163, PE-labelled anti-mouse CD86, respectively (BioLegend). After washed for three times, the proportion of cells and the fluorescence intensity of TAMs were measured by flow cytometry, using an isotype-matched control antibody for background subtraction. Samples were further analyzed on BD LSR II instrument (BD Biosciences, USA).

### Animal experiments

BALB/c nude mice (male, 6–8 weeks, 18–22 g) were obtained from Beijing Vital River Laboratory Animal Technology Co. Ltd., Beijing, China. All mouse experiments were conducted in accordance with the guidelines of the Laboratory Animal Welfare and Ethics Committee of Third Military Medical University, approval number: AMUWEC20191575. Tumor model was constructed by subcutaneously inoculating 1 × 10^6^ PC3 cells suspension in 150 µL cell culture medium into the left back of mice. The mice were used for further experiments when the tumor volumes reached about 100 mm^3^, which was about 3 weeks after tumor inoculation.

To investigate the impact of RMnMels-mediated macrophage polarization and hyperthermia therapy on tumor growth, PC3 tumor-bearing mice were randomly assigned to five groups: PBS (group I), PBS plus 690 nm laser irradiation (group II), RMnMels only (group III), PMnMels plus 690 nm laser irradiation (group IV), and RMnMels plus 690 nm laser irradiation (group V). The mice in group III, IV and V were intravenously injected with nanocomposites with 12 mg/kg BW. At 6 h post injection, the tumor sites were irradiated by a 690 nm laser at 500 mW/cm^2^ for 15 min. Thermal images at the tumor sites were obtained using a FLIR E75-42 Advanced Thermal Imaging Camera. The tumor sizes were measured every two days and the tumor volumes were calculated as volume = (length × width × width)/2. The body weight and tumor growth profiles were monitored for a period of 19 days post treatment, and then the mice were euthanized and their tumor tissues were collected for further analysis.

### Serum biochemical measurement

Blood was collected from tumor-bearing mice on day 19 post treatment and healthy mice at different time points (12 mg/kg BW). Mice that received PBS treatment were set as control. To obtain serum, fresh ocular vein blood samples were harvested from mice, then centrifuged for three times at a speed of 3500 rpm for 15 min. The serum biochemical indicators were measured using an automatic biochemical analyzer (Cobas 8000, Roche, Germany).

### Histological analysis

Hematoxylin and eosin (H&E) staining, TUNEL and Ki-67 immunohistochemical staining were performed according to standard procedures. The mice were euthanized on the 19th day after different treatments, and their main organs were harvested and fixed in 4% formalin overnight. The samples were embedded and sectioned at a thickness of 5 μm. The sections were stained and images were taken using 3D HISTECH Pannoramic 250 (3DHISTECH, Hungary).

## Supplementary Information

**Additional file 1: Figure S1.** EDX analysis of RMnMels. **Figure S2.** (a)XPS survey spectra. XPS spectra of (b) C1s, (c) N1s and (d) O1s for RMnMels. **Figure S3.** (a) Hydrodynamicsizes of nanocomposites. (b) Zeta potential of nanocomposites. **Figure S4.** The linear relationship for the *r*_1_relaxivity of RMnMels versus Mnconcentrations. The insets show corresponding *T*_1_-weighted MRimages. **Figure S5.** (a) *T*_1_-weightedMR images of mice kidneys and liver prior to and at various time points postintravenous injection of RMnMels at 12 mg/kg body weight. The images wereacquired at 1.5 T.Normalized signal intensities determined from mice (b) kidneys and (c) liver. **Figure S6.** UV–vis absorption profile ofRMnMels with various mass concentrations. **Figure S7.** Invitro photoacoustic signal intensities of RMnMels versus solution mass concentrations.The insets show corresponding photoacoustic images. **Figure S8.** The amount of manganese (Mn) elements per gramof tissue. **Figure S9.** Cell viability ofPC3 and RAW 264.7 cells after co-incubation with various concentrations ofRMnMels. **Figure S10.** (a) Optical microscope images of RAW 264.7cells after co-incubation with various concentrations of RMnMels for 12 h. Scale bars, 100 µm. (b) Opticalmicroscope images of PC3 cells after incubation without (upper) or with (lower)50 µg/mL RMnMels for 12 h. Scale bars, 100 µm. **FigureS11.** (a) Cellular TEM images ofPC3 cells after incubation without (upper) or with (lower) RMnMels at 50 µg/mLfor 12 h. (b) Tissue TEM images of PC3 tumor tissue slices after intravenous injection of PBS(upper) or RMnMels (lower) for 48 h. **Figure S12.** Cellular TEM imagesof lipopolysaccharide induced M1-like macrophage after incubation withRMnMels at 50 µg/mL for 12 h. **Figure S13.** (a) TEM images ofRMnMels with/without 2.5 mM H_2_O_2_ for 72 h. Scale bar, 200 nm.The insets show corresponding digital photographs. (b) UV–vis absorption spectra of RMnMels co-incubation with 2.5 mM H_2_O_2_ atdifferent time points. **Figure S14.** (a) Infrared thermalimages and (b) temperaturevariations of 200 μg/mL RMnMels aqueous solution during exposure to a 690 nm laser with different powerdensities for 300 s. (c) Infrared thermal images and (d) temperature variations of RMnMels aqueous solution with differentconcentrations during exposure to a 690 nm laser at 500 mW/cm^2^for 300 s. **Figure S15.** Photothermal heating curvesof RMnMels (100 μg/mL) irradiated by 690 nm laser at 500 mW/cm^2^ over three laser ON/OFF cycles. **FigureS16.** (a) TEM images of RMnMels solution before and after 690 nm laserirradiation (500 mW/cm^2^, 30 min). Scale bar, 200 nm. The insets showcorresponding digital photographs. (b) UV–vis absorption spectra of RMnMels aqueoussolutions prior to and after exposure to 690 nm laser irradiation. **FigureS17.** Flow cytometric assay of the polarization ofRAW 264.7 cells toward M1 phenotype. **Figure S18.** Body weight curves of PC3 tumor-bearing mice during a periodof 19 days after different treatments. **FigureS19.** Flow cytometry analysis of tumor immune cells. Gating strategy showingdelineation of (a) singlets cells. (b) numbers indicate the percentages of thecells within the gates. (c) live cells. (d) CD45^+^ leukocytes. (e) tumor-associatedmacrophages. **Figure S20.** H&Estained images of mice major organ sections on day 19 after differenttreatments. Scale bar, 100 µm. **FigureS21.** Serum biochemical indexes of healthy mice obtained on different timepoint. **Table S1.** Primer sequences for different genes.

## Data Availability

All data used to generate these results is available in the main text and supporting information.
