# Supplementary material for: Biomimetic manganese-eumelanin nanocomposites for combined hyperthermia-immunotherapy against prostate cancer
Source: J Nanobiotechnology. 2022 Jan 24;20:48. doi: 10.1186/s12951-022-01248-5 (PMC8785565; doi:10.1186/s12951-022-01248-5)
Supplement: Supplementary file 1 — Additional file 1: Figure S1. EDX analysis of RMnMels. Figure S2. (a)XPS survey spectra. XPS spectra of (b) C1s, (c) N1s and (d) O1s for RMnMels. Figure S3. (a) Hydrodynamicsizes of nanocomposites. (b) Zeta potential of nanocomposites. Figure S4. The linear relationship for the r1relaxivity of RMnMels versus Mnconcentrations. The insets show corresponding T1-weighted MRimages. Figure S5. (a) T1-weightedMR images of mice kidneys and liver prior to and at various time points postintravenous injection of RMnMels at 12 mg/kg body weight. The images wereacquired at 1.5 T.Normalized signal intensities determined from mice (b) kidneys and (c) liver. Figure S6. UV–vis absorption profile ofRMnMels with various mass concentrations. Figure S7. Invitro photoacoustic signal intensities of RMnMels versus solution mass concentrations.The insets show corresponding photoacoustic images. Figure S8. The amount of manganese (Mn) elements per gramof tissue. Figure S9. Cell viability ofPC3 and RAW 264.7 cells after co-incubation with various concentrations ofRMnMels. Figure S10. (a) Optical microscope images of RAW 264.7cells after co-incubation with various concentrations of RMnMels for 12 h. Scale bars, 100 µm. (b) Opticalmicroscope images of PC3 cells after incubation without (upper) or with (lower)50 µg/mL RMnMels for 12 h. Scale bars, 100 µm. FigureS11. (a) Cellular TEM images ofPC3 cells after incubation without (upper) or with (lower) RMnMels at 50 µg/mLfor 12 h. (b) Tissue TEM images of PC3 tumor tissue slices after intravenous injection of PBS(upper) or RMnMels (lower) for 48 h. Figure S12. Cellular TEM imagesof lipopolysaccharide induced M1-like macrophage after incubation withRMnMels at 50 µg/mL for 12 h. Figure S13. (a) TEM images ofRMnMels with/without 2.5 mM H2O2 for 72 h. Scale bar, 200 nm.The insets show corresponding digital photographs. (b) UV–vis absorption spectra of RMnMels co-incubation with 2.5 mM H2O2 atdifferent time points. Figure S14. (a) Infrared thermalimages [file 12951_2022_1248_MOESM1_ESM.docx]

Additional file 1

Biomimetic Manganese-eumelanin Nanocomposites for Combined Hyperthermia-immunotherapy against Prostate Cancer

Yu Liu,^1,2,3,5‡^ Wenting Shang,^3‡^ Heng Liu,^4‡^ Hui Hui^3^, Jun Wu^3^, Wei Zhang^3^, Pengli Gao^1,3,5^, Kunxiong Guo^3^, and Yanli Guo^2^*, Jie Tian^1,3,5,6^*

^‡^These authors contributed equally to this work.

Dr. Y. Liu, Dr. P. Gao, Prof. J. Tian

^1^Beijing Advanced Innovation Center for Big Data-Based Precision Medicine, School of Medicine and Engineering, Beihang University, Beijing, 100191, China

Dr. Y. Liu, Prof. Y. Guo

^2^Department of Ultrasound, Southwest Hospital, Army Medical University, Chongqing 400038, China

Dr. W. Shang, Dr. H. Hui, Dr. J. Wu, Dr. W. Zhang, M.M. K. Guo

^3^CAS Key Laboratory of Molecular Imaging, Beijing Key Laboratory of Molecular Imaging, the State Key Laboratory of Management and Control for Complex Systems, Institute of Automation, Chinese Academy of Sciences, Beijing, 100190, China

Dr. H. Liu

^4^Department of Radiology, PLA Rocket Force Characteristic Medical Center, Beijing 100088, China

Dr. Y. Liu, Dr. P. Gao, Prof. J. Tian

^5^Key Laboratory of Big Data-Based Precision Medicine (Beihang University), Ministry of Industry and Information Technology, Beijing, 100191, China

Prof. J. Tian

^6^Engineering Research Center of Molecular and Neuro Imaging of Ministry of Education, School of Life Science and Technology, Xidian University, Xi’an, Shaanxi, 710126, China

Corresponding author: Yanli Guo (guoyanli71@aliyun.com); Jie Tian ([jie.tian@ia.ac.cn](mailto:jie.tian@ia.ac.cn))

**Figure S1.** EDX analysis of RMnMels.

**Figure S2.** (a) XPS survey spectra. XPS spectra of (b) C1s, (c) N1s and (d) O1s for RMnMels.

The spectral peak was calibrated with the C-C /C-H energy position of C1s adsorbed carbon at 284.8eV.

**Figure S3.** (a) Hydrodynamic sizes of nanocomposites. (b) Zeta potential of nanocomposites.

**Figure S4.** The linear relationship for the *r*_1_ relaxivity of RMnMels *versus* Mn concentrations. The insets show corresponding *T*_1_-weighted MR images.

**Figure S5.** (a) *T*_1_-weighted MR images of mice kidneys and liver prior to and at various time points post intravenous injection of RMnMels at 12mg/kg body weight. The images were acquired at 1.5 T. Normalized signal intensities determined from mice (b) kidneys and (c) liver.

**Figure S6.** UV-vis absorption profile of RMnMels with various mass concentrations.

**Figure S7.** *In vitro* photoacoustic signal intensities of RMnMels *versus* solution mass concentrations. The insets show corresponding photoacoustic images.

**Figure S8**. The amount of manganese (Mn) elements per gram of tissue.

**Figure S9.** Cell viability of PC3 and RAW 264.7 cells after co-incubation with various concentrations of RMnMels.

**Figure S10.** (a) Optical microscope images of RAW 264.7 cells after co-incubation with various concentrations of RMnMels for 12 h. Scale bars, 100 µm. (b) Optical microscope images of PC3 cells after incubation without (upper) or with (lower) 50 µg/mL RMnMels for 12 h. Scale bars, 100 µm.

**Figure S11.** (a) Cellular TEM images of PC3 cells after incubation without (upper) or with (lower) RMnMels at 50 µg/mL for 12 h. (b) Tissue TEM images of PC3 tumor tissue slices after intravenous injection of PBS (upper) or RMnMels (lower) for 48 h.

**Figure S12.** Cellular TEM images of lipopolysaccharide induced M1-like macrophage after incubation with RMnMels at 50 µg/mL for 12 h.

**Figure S13.** (a) TEM images of RMnMels with/without 2.5 mM H_2_O_2_ for 72 h. Scale bar, 200 nm. The insets show corresponding digital photographs. (b) UV-vis absorption spectra of RMnMels co-incubation with 2.5 mM H_2_O_2_ at different time points.

**Figure S14.** (a) Infrared thermal images and (b) temperature variations of 200 μg/mL RMnMels aqueous solution during exposure to a 690 nm laser with different power densities for 300 s. (c) Infrared thermal images and (d) temperature variations of RMnMels aqueous solution with different concentrations during exposure to a 690 nm laser at 500 mW/cm^2^ for 300 s.

**

**

**Figure S15**. Photothermal heating curves of RMnMels (100 ug mL^-1^) irradiated by 690 nm laser at 500 mW/cm^2^ over three laser ON/OFF cycles.

**Figure S16.** (a) TEM images of RMnMels solution before and after 690 nm laser irradiation (500 mW/cm^2^, 30 min). Scale bar, 200 nm. The insets show corresponding digital photographs. (b) UV-vis absorption spectra of RMnMels aqueous solutions prior to and after exposure to 690 nm laser irradiation.

**Figure S17.** Flow cytometric assay of the polarization of RAW 264.7 cells toward M1 phenotype.

**Figure S18**. Body weight curves of PC3 tumor-bearing mice during a period of 19 days after different treatments.

**Figure S19**. Flow cytometry analysis of tumor immune cells. Gating strategy showing delineation of (a) singlets cells. (b) numbers indicate the percentages of the cells within the gates. (c) live Cells. (d) CD45^+^ leukocytes. (e) tumor-associated macrophages.

**Figure S20**. H&E stained images of mice major organ sections on day 19 after different treatments. Scale bar, 100 µm.

**Figure S21**. Serum biochemical indexes of healthy mice obtained on different time point.

**Table S1** Primer sequences for different genes.

| Gene name | Forward primer | Reverse primer | |  |
| --- | --- | --- | --- | --- |
| TNF-α | TCCCCAAAGGGATGAGAAGTT | | GAGGAGGTTGACTTTCTCCTGG | |
| CD86 | CTAAGCAAGGTCACCCGAAAC | | GGCCACAGTAACTGAAGCTGTAT | |
| iNOS | CATTCAGATCCCGAAACGCT | | TGTAGGACAATCCACAACTCGC | |
| CD206 | AGCTGCTGTCGCTGGAAT | | GGATGCTTGAGAAGTGAATAGG | |
| Arginase-1 | TGTGGGAAAAGCCAATGAA | | GGTGTCAGCGGAGTGTTG | |
| GAPDH | TGAAGGGTGGAGCCAAAAG | | AGTCTTCTGGGTGGCAGTGAT | |
